# Graphene Monolayer Nanomesh Structures and Their Applications in Electromagnetic Energy Harvesting for Solving the Matching Conundrum of Rectennas

**DOI:** 10.3390/nano14191542

**Published:** 2024-09-24

**Authors:** Mircea Dragoman, Adrian Dinescu, Martino Aldrigo, Daniela Dragoman, Elaheh Mohebbi, Eleonora Pavoni, Emiliano Laudadio

**Affiliations:** 1 National Institute for Research and Development in Microtechnologies (IMT), 077190 Voluntari, Ilfov, Romania; mircea.dragoman@imt.ro (M.D.); adrian.dinescu@imt.ro (A.D.); 2 Physics Faculty, University of Bucharest, P.O. Box MG-11, 077125 Bucharest, Romania; daniela.dragoman@unibuc.ro; 3 Academy of Romanian Scientists, Strada Ilfov, Nr. 3, 050044 Bucharest, Romania; 4 Department of Science and Engineering of Matter, Environment and Urban Planning, Marche Polytechnic University, Via Brecce Bianche, 60131 Ancona, Italy; e.mohebbi@staff.univpm.it (E.M.); e.pavoni@staff.univpm.it (E.P.); e.laudadio@staff.univpm.it (E.L.)

**Keywords:** diode, electromagnetic radiation, energy harvesting, graphene, quantum technologies

## Abstract

In this paper, we investigate various graphene monolayer nanomesh structures (diodes) formed only by nanoholes, with a diameter of just 20 nm and etched from the graphene layer in different shapes (such as rhombus, bow tie, rectangle, trapezoid, and triangle), and their electrical properties targeting electromagnetic energy harvesting applications. In this respect, the main parameters characterizing any nonlinear device for energy harvesting are extracted from tens of measurements performed on a single chip containing the fabricated diodes. The best nano-perforated graphene structure is the triangle nanomesh structure, which exhibits remarkable performance in terms of its characteristic parameters, e.g., a 420 Ω differential resistance for optimal impedance matching to an antenna, a high responsivity greater than 10^3^ V/W, and a low noise equivalent power of 847 pW/√Hz at 0 V.

## 1. Introduction

Graphene monolayers, which are single sheets of carbon atoms organized in a honeycomb lattice, show an impressive electrical performance, such as high mobility or ballistic transport at room temperature with a mean free path of hundreds of nanometers, but with no bandgap [1]. When a graphene monolayer is perforated with an array of holes termed as antidots [2] or nanomeshes, a bandgap of 200–300 meV is opened in the graphene [3,4,5].

In the case of field-effect transistors (FETs), whose channel is a graphene monolayer perforated with nanoholes (with a diameter of 20 nm and a period of 100 nm), and with a channel width of 2 µm and different channel lengths between 1 and 8 µm, the mobility attains values from 10,400 cm^2^/V∙s for a channel length of 1 µm to about 550 cm^2^/V∙s for a channel length of 8 µm. All of these transistors have an on–off ratio of at least 10^3^ at drain and gate voltage values less than 2 V. Therefore, by changing the length of the nano-perforated graphene channel, we are able to engineer the mobility of the carriers, thus making it similar to that of Si, GaAs, or InP [5].

The applications of nano-perforated graphene monolayers are found in many nanotechnology areas, such as biosensors [6], gas sensors [7], pressure sensors [8], and electronic skin [9]. In particular, nanomeshes have shown amazing results in low thermal conductivity [10] and thermoelectric applications [11]. Graphene nanomeshes are also used as transparent electrodes for solar cells [12]. Very recently, FETs possessing a graphene nanomesh channel have been used to harvest microwaves using the pyroelectric effect at room temperature [13] and at cryogenic temperatures [14]. There are important applications of the nano-perforated (or holey) graphene in batteries, supercapacitors, and other energy storage devices [15]. Hence, it is straightforward to investigate whether graphene nanomeshes can be used for electromagnetic energy harvesting applications.

Electromagnetic energy harvesting from microwaves to infrared radiation can be performed by an antenna with a nonlinear element (which could be an unbiased diode or transistor [16,17]) integrated between its two arms, called a rectenna. The incoming electromagnetic radiation is transformed into a DC signal by the nonlinear device. We have to point out that the current–voltage dependence of the nonlinear element could not resemble a typical rectification diode, in which there is a region of very low current followed by a region where the current rapidly increases by two or three orders of magnitude. For example, a well-known nonlinear device suitable for electromagnetic energy harvesting from microwaves to infrared is the metal–insulator–metal (MIM) diode, a tunnelling diode where the current has an exponential dependence on voltage irrespective of the fact that the latter is either positive or negative [18].

In this work, we have fabricated at the wafer level and measured uniform-shaped (rectangle) and nonuniform-shaped (triangle, trapezoid, rhombus, bow tie) structures made of graphene monolayers, transferred on a 4-inch wafer of doped silicon/silicon dioxide (Si/SiO_2_), and formed exclusively by nanoholes (with a diameter of 20 nm). Then, we have extracted the performance parameters described above for evaluating the best geometry suitable for electromagnetic energy harvesting applications. The most promising configuration is the “triangle” nanomesh diode, for which ab initio calculations are provided to delve into the transport mechanism at the quantum level. The atomistic simulations are in excellent agreement with the measurements, thus proving that quantum-confined structures like graphene nanoribbons and quantum dots or antidots, i.e., nanomeshes, allow for the opening of a bandgap in the graphene, hence drastically reducing the metal–graphene resistance.

## 2. Key Performance Parameters of a Diode and Their Extraction from the Current–Voltage Characteristics

Regardless of the nonlinear device used in the rectenna, there are several performance parameters that can be extracted from the current–voltage characteristics which characterize any device used in electromagnetic energy harvesting, such as diodes. These parameters are as follows:

the differential resistance, defined as

R_D_ = 1⁄(∂I⁄∂V), (1)

the nonlinearity, defined as

χ = (∂I⁄∂V)⁄(I⁄V),(2)

the sensitivity, defined as

γ = (∂^2^I⁄∂V^2^)⁄(∂I⁄∂V), (3)

the responsivity, defined as

β = 0.5 R_D_ γ, (4)

and the noise equivalent power, defined as

NEP = √(4 k_B_ T R_D0_)⁄β, (5)

where R_D0_ = R_D_ (V = 0) is the differential resistance R_D_ (in Ω) at 0 V. While the nonlinearity, the sensitivity, and the responsivity must be as high as possible, the differential resistance and the noise equivalent power must attain minimum values to ensure an efficient conversion of the electromagnetic energy into DC power. In fact, minimizing R_D_ allows for optimal impedance matching to an antenna, which is a prerequisite for the maximum power transfer to the nonlinear device. At the same time, it is desirable to have the NEP as low as possible, since a low NEP value corresponds to a lower noise floor, which ensures a more sensitive harvester and lower noise characteristics in the output signal.

## 3. Fabrication of the Graphene Nanomesh Structures at the Wafer Scale 

**Figure 1:**
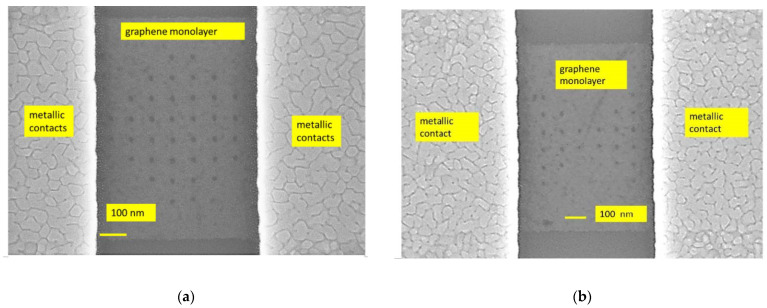
Graphene monolayer nanomesh structures with metallic contacts: (**a**) rhombus and (**b**) bow tie.

**Figure 2:**
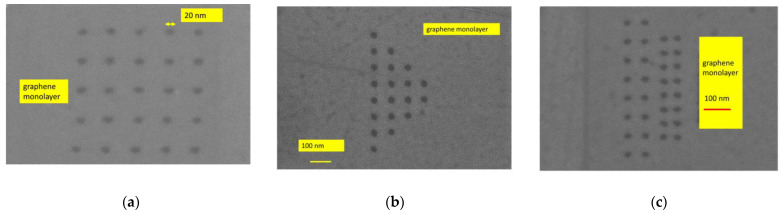
Details of graphene monolayer nanomeshes of different shapes: (**a**) rectangle, (**b**) triangle, and (**c**) delta.

**Figure 3:**
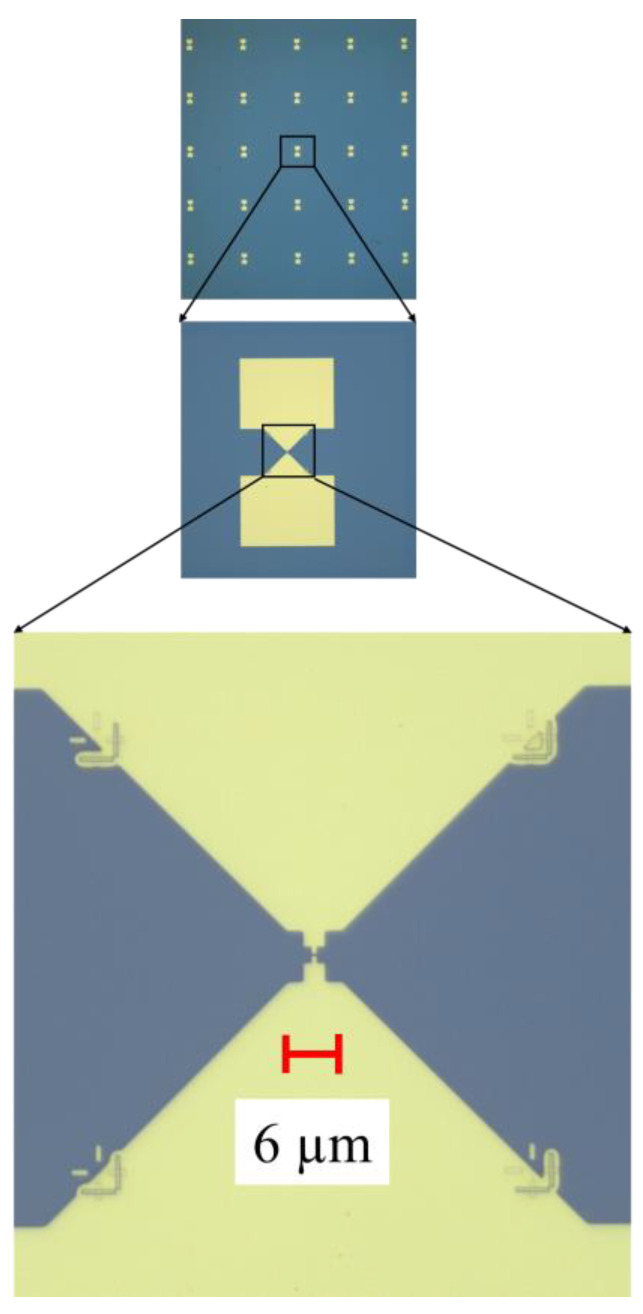
A part of the graphene nanomesh chip with metallized electrodes and its details.

The uniform and nonuniform graphene nanomesh devices were fabricated on a doped Si/SiO_2_ 4-inch wafer. The thickness of the Si wafer is 525 µm and the SiO_2_ layer has a thickness of 300 nm. The SiO_2_ was grown by thermal oxidation. Further, the graphene monolayer was transferred onto the Si/SiO_2_ substrate by Graphenea (San Sebastian, Spain). The technological processes, as well as a review of the fabrication methods of the graphene nanomesh used by us, are given in detail elsewhere and will be only briefly explained here [15,19]. Raman spectroscopy was used to map the graphene monolayer chip. We have observed that about 80% of the wafer’s surface was covered with the graphene monolayer, the rest being areas with graphene multilayers containing 4–6 monolayers and grain boundary defects. The fabrication consisted of the following main steps: (i) patterning the graphene channel by electron-beam lithography (EBL) and reactive ion etching (RIE); (ii) patterning the shapes of the nanomesh geometries using EBL; (iii) patterning, metallization, and liftoff of the metallic contacts, i.e., Cr (5 nm)/Au (240 nm) deposited using an e-beam process (the chrome thin film being necessary as an adhesion layer for gold). In total, 100 graphene nanomesh devices were measured. In Figure 1, Figure 2 and Figure 3, we show the SEM images of the graphene nanomesh devices of different forms. In Figure 1a,b, the rhombus and bow-tie graphene nanomesh structures with their metallic contacts are displayed. In Figure 2a–c, we present in more detail several graphene monolayer nanomeshes in the shape of a rectangle (Figure 2a), a triangle (Figure 2b), and a trapezoid (Figure 2c, further termed as “delta”) without metal contacts. Finally, Figure 3 shows part of the fabricated wafer and details of the metallic contacts at an increasing degree of magnification.

## 4. Measurements and Discussion

The current–voltage measurements were made using Keithley SCS 4200 equipment (Keithley Instruments, Inc., Cleveland, OH, USA). All measurements were made at room temperature. The entire probe station for on-wafer characterization was located inside a Faraday cage and connected to the equipment via low-noise amplifiers. All of the 100 devices were measured and 90 of them worked with a remarkable reproducibility, since the current measurements at the same voltage of the same kind of graphene nanomesh structures exhibit variances of less than 3%. 

We have extracted the performance parameters using Equations (1)–(5) described above, i.e., the differential resistance R_D_ (Ω), the nonlinearity χ (a.u.), the sensitivity γ (V^−1^), the voltage responsivity β (V/W), and the noise equivalent power NEP (pW/√Hz), with all of them in the voltage range between −1 and 1 V. We present these parameters for all of the types of graphene nanomesh structures fabricated as follows: rhombus, bow tie, rectangle (two versions of it, i.e., with a distance between the nanoholes of 70 nm and 100 nm, respectively), triangle, and delta.

**Figure 4:**
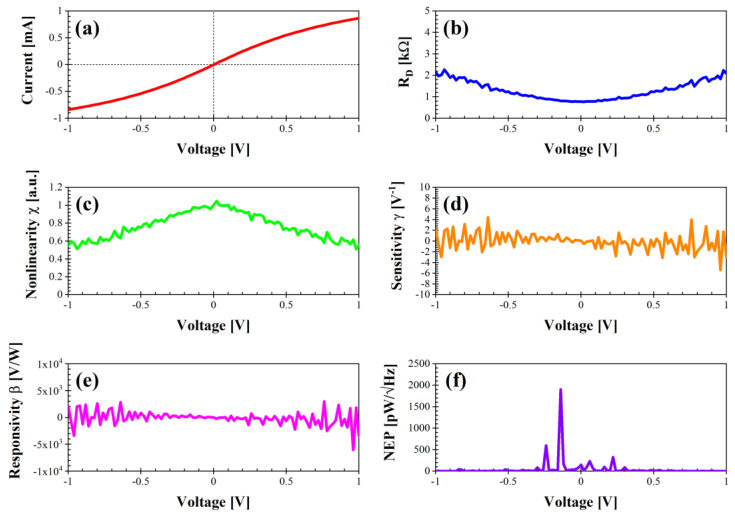
Performance of diode “bow tie” in dark conditions: (**a**) I–V characteristic; (**b**) differential resistance R_D_ (kΩ); (**c**) nonlinearity χ (a.u.); (**d**) sensitivity γ (V^−1^); (**e**) voltage responsivity β (V/W); (**f**) noise equivalent power (pW/√Hz).

Figure 4 depicts the performance of the nanomesh bow-tie graphene structure. The DC current is quite high and attains values between approximately −0.81 and 0.86 mA, with a minimum R_D_ of 0.77 kΩ at 0 V and a maximum χ = 1 at 0.02 V. The sensitivity spans the range between −4 and 4 V^−1^ (0.06 V^−1^ at 0 V), with an excellent responsivity between −5 × 10^3^ and 5 × 10^3^ V/W (24 V/W at 0 V), whereas the maximum NEP is 1900 pW/√Hz at −0.14 V and NEP = 150 pW/√Hz at 0 V. We note here that a negative voltage responsivity is associated with an n-type behavior, whereas a p-type behavior generates a positive voltage responsivity [20]. From Figure 4, it is apparent that this device is an excellent candidate for zero-bias detection/energy harvesting, especially considering the acceptable value of its differential resistance (which can reach several kΩ for graphene geometric diodes, thus making the design of a matching network for maximum power transfer almost impossible).

**Figure 5:**
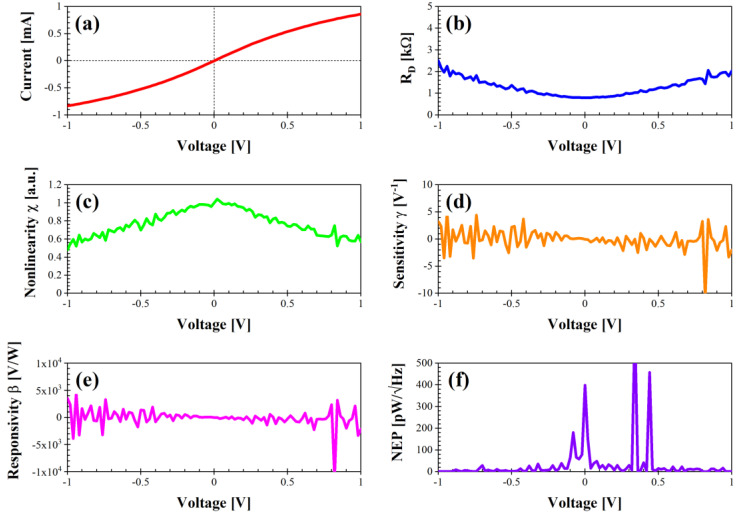
Performance of diode “rhombus” in dark conditions: (**a**) I–V characteristic; (**b**) differential resistance R_D_ (kΩ); (**c**) nonlinearity χ (a.u.); (**d**) sensitivity γ (V^−1^); (**e**) voltage responsivity β (V/W); (**f**) noise equivalent power (pW/√Hz).

The performance of the diode with the shape of a rhombus (Figure 5) is similar to that of the graphene nanomesh structure with the shape of a bow tie in terms of current level, R_D_ (0.79 kΩ at 0 V), nonlinearity, and responsivity (in this case, the minimum is about −1 × 10^4^ and β = −9 V/W at 0 V), but the maximum NEP is substantially lower (457 pW/√Hz at 0.44 V). However, NEP = 398 pW/√Hz at 0 V, and on average, the NEP is higher than that of the bow-tie graphene nanomesh structure.

**Figure 6:**
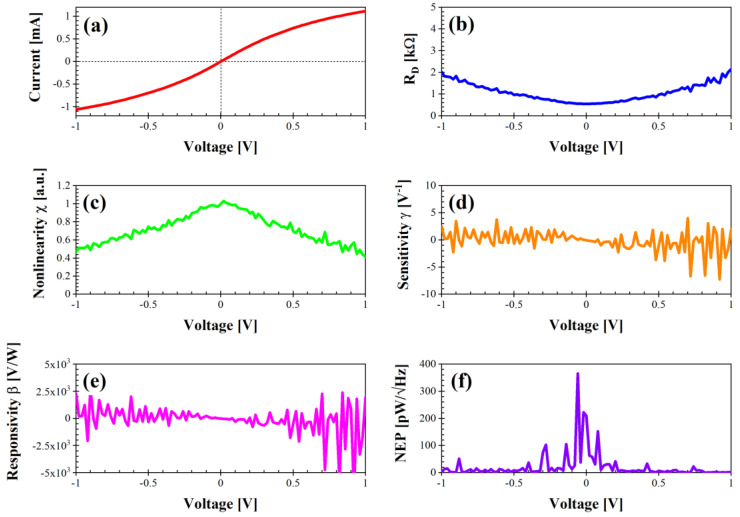
Performance of diode “delta” in dark conditions: (**a**) I–V characteristic; (**b**) differential resistance R_D_ (kΩ); (**c**) nonlinearity χ (a.u.); (**d**) sensitivity γ (V^−1^); (**e**) voltage responsivity β (V/W); (**f**) noise equivalent power (pW/√Hz).

The graphene nanomesh structure with the trapezoid shape and termed as “delta” (Figure 6) shows R_D_ = 0.55 kΩ at 0 V, with similar values for the nonlinearity to the rhombus graphene nanomesh structure, a slightly higher sensitivity (−0.05 V^−1^ at 0 V), a responsivity with the maximum around 2.5 × 10^3^ V/W (−14.25 V/W at 0 V), and a low NEP (the maximum is 365 pW/√Hz at −0.06 V). Hence, this delta-shaped diode guarantees an even easier impedance matching in low-power high-frequency applications working at voltage values near 0 V, as well as a very low NEP.

**Figure 7:**
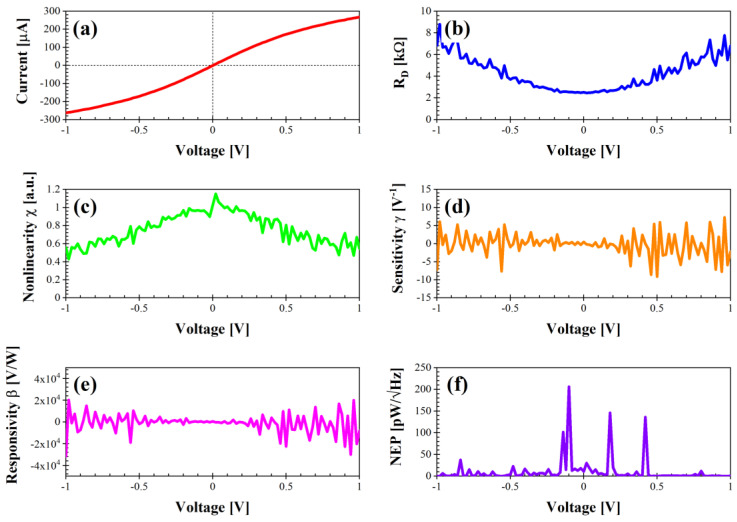
Performance of diode “rectangle#1” in dark conditions: (**a**) I–V characteristic; (**b**) differential resistance R_D_ (kΩ); (**c**) nonlinearity χ (a.u.); (**d**) sensitivity γ (V^−1^); (**e**) voltage responsivity β (V/W); (**f**) noise equivalent power (pW/√Hz).

**Figure 8:**
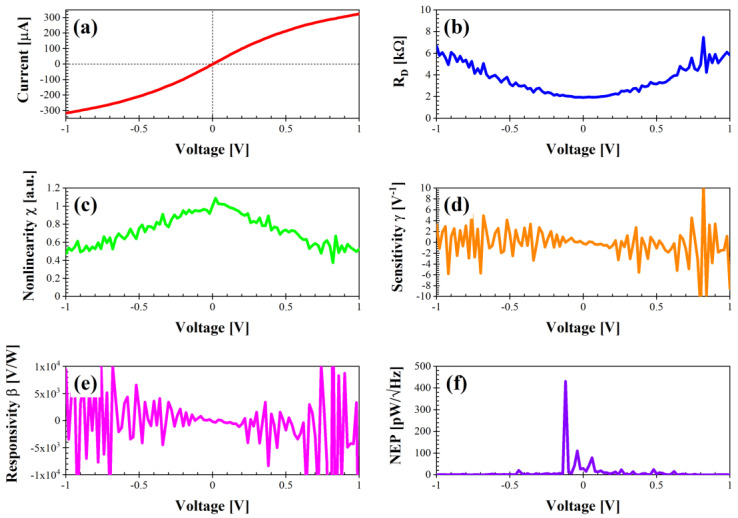
Performance of diode “rectangle#2” in dark conditions: (**a**) I–V characteristic; (**b**) differential resistance R_D_ (kΩ); (**c**) nonlinearity χ (a.u.); (**d**) sensitivity γ (V^−1^); (**e**) voltage responsivity β (V/W); (**f**) noise equivalent power (pW/√Hz).

If we now consider the two rectangle-shaped diodes termed as “rectangle#1” and “rectangle#2” (Figure 7 and Figure 8, respectively), one can notice the much lower current values (between −300 and 300 µA). Consequently, R_D_ is also higher at 0 V: 2.5 kΩ for the diode “rectangle#1” and 1.92 kΩ for the diode “rectangle#2”. However, the nonlinearity is the highest among all of the fabricated diodes, 1.15 at 0.02 V for the diode “rectangle#1” and 1.09 at 0.02 V for the diode “rectangle#2”, together with a higher sensitivity between −10 and 10 V^−1^. The responsivity is also higher, i.e., in the range −3 × 10^4^–2 × 10^4^ for the diode “rectangle#1” and in the range −1 × 10^4^–1 × 10^4^ for the diode “rectangle#2”. The NEP is particularly low for the diode “rectangle#1” and does not exceed 206 pW/√Hz at −0.1 V, while for the diode “rectangle#2” the behavior is quite homogeneous despite the peak of 431 pW/√Hz at −0.12 V. Hence, these two diodes are suitable for low-power and low-noise applications requiring a high sensitivity and a high responsivity, at the cost of some matching issues.

**Figure 9:**
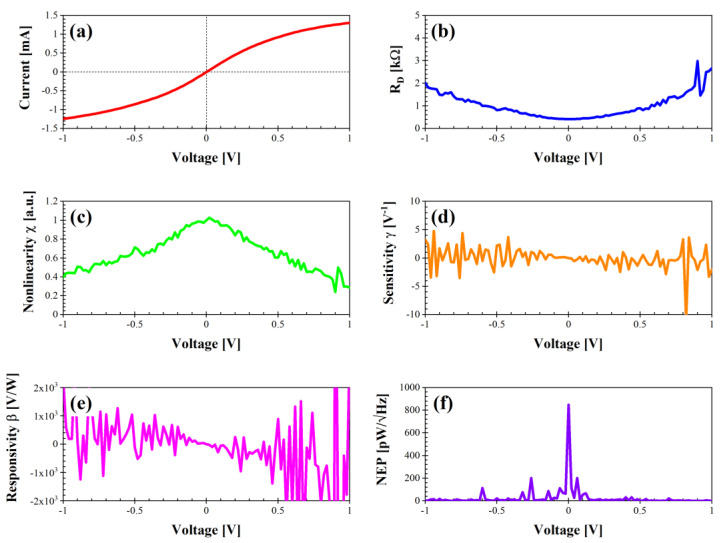
Performance of diode “triangle” in dark conditions: (**a**) I–V characteristic; (**b**) differential resistance R_D_ (kΩ); (**c**) nonlinearity χ (a.u.); (**d**) sensitivity γ (V^−1^); (**e**) voltage responsivity β (V/W); (**f**) noise equivalent power (pW/√Hz).

Finally, we report here the performance of the graphene nanomesh structure with the shape of a triangle (Figure 9). At first glance, its parameters resemble those obtained for the bow tie and rhombus, but the DC current is the highest (between −1.24 and 1.30 mA) and R_D_ is the lowest (i.e., 0.42 kΩ at 0 V), thus making it the most suitable for integration with an antenna. The nonlinearity, sensitivity, and responsivity are similar to the bow-tie and rhombus graphene nanomesh structures. Last, the NEP has a peak of 847 pW/√Hz at 0 V but it attains quite low values over the whole voltage range.

The question that immediately arises is which is the best structure for electromagnetic energy harvesting? The triangle-shaped graphene nanomesh structure is the best one because it exhibits the lowest R_D_ of only 420 Ω, meaning that it can be easily matched to an antenna like a bow tie, which has an input impedance in the range of 200–300 Ω. This result is extremely significant and solves a quite important conundrum, if one considers that the impedance matching to an antenna is a major issue for electromagnetic energy harvesting, since it hinders the maximum power transfer to the rectifying diode. With this type of structure, we are far from the performance of uniform graphene monolayers with a microwave impedance of around 2–3 kΩ, this aspect being the main reason why graphene antennas have very low radiation efficiency values in microwaves and millimeter waves. Moreover, if we compare our results with similar ones obtained for various types of nonlinear devices dedicated to electromagnetic energy harvesting in microwaves (and shown in Tables III and IV in [21]), we see that the proposed graphene monolayer nanomesh structures are by far among the best fabricated and measured up to now, especially regarding the differential resistance, i.e., the most critical parameter for matching a nonlinear device to an antenna. 

Why? It is notorious that the graphene/metal contact resistance is high due to the lack of a bandgap in graphene monolayers; nevertheless, there are numerous methods to reduce it as reported in many review papers [22,23]. Thus, the solution is to open a bandgap in the graphene to reduce the contact resistance. The quantum-confined structures like graphene nanoribbons and quantum dots or antidots, i.e., nanomeshes, allow for the opening of a bandgap in the graphene, hence drastically reducing the metal–graphene resistance [22]. In fact, nanomesh structures can be classified as a type of semiconducting graphene that facilitates the alignment of the Fermi levels between the metal of the contact and the graphene itself. This can be seen from any R_D_(V) dependence, which can be easily approximated as a linear one.

Finally, we have shown in [13] that an FET with a graphene monolayer channel with a length L_ch_ = 2500 nm and a width W_ch_ = 2000 nm, perforated with holes with a diameter of 30 nm and a distance of 100 nm along the horizontal and vertical axes (hence, a very large nanomesh rectangle, much larger than what has been presented here), is able to work as a microwave detector/harvester up to 10 GHz at room temperature. The graphene nanomesh structures of this work possess a length that is at least three to four times smaller, whereas the width is two times smaller. For this reason, we can expect that these devices could work as harvesters at frequencies as high as 40 GHz or even more. All of these nano-perforated devices are a part of the so-called graphene nanoporous materials, with excellent absorbing properties at microwaves and at infrared frequencies [3].

## 5. Ab Initio Simulations of the “Triangle” Nanomesh Diode

To further assess the transport mechanism at the quantum level of the “triangle” nanomesh diode and, hence, demonstrate the opening of a bandgap in the nanopatterned graphene, we performed ab initio simulations as described in the following.

**Figure 10:**
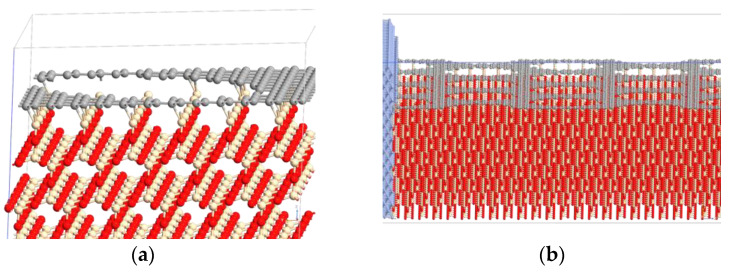

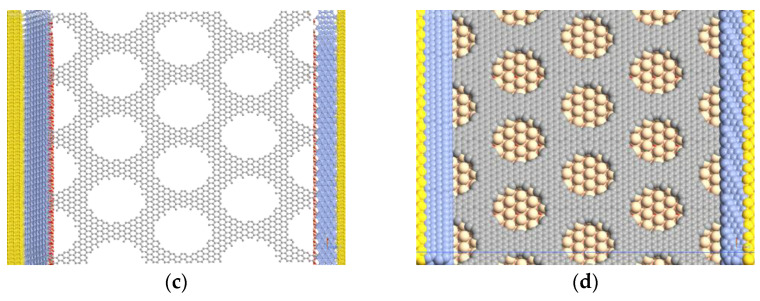
Optimized perforated graphene–SiO_2_ interface: (**a**) focus on Si-C bonds and (**b**) wavy shape of interface; (**c**) top view of perforated graphene and (**d**) top view of holes. C, Si, and O atoms are highlighted in gray, light brown, and red, respectively, whereas blue and yellow represent Cr and Au atoms, respectively.

The device has been modeled at an atomistic level in each of its components by means of an approach similar to that described in [24]. In order to obtain a diode with a geometry close to the fabricated one, the interfaces generated by the various components have been deeply investigated. First, the SiO_2_–graphene interface has been analyzed, and from Density Functional Theory (DFT) studies, it has been determined that the terminal Si atoms of cristobalite generate strong covalent bonds with the C atoms of graphene; on the contrary, when analyzing the opposite interface, the hydroxylated regions of SiO_2_ are able to create only weak van der Waals interactions with the graphene, thus making this geometry not plausible in a real device. This assumption is also in line with another work in the literature investigating the SiO_2_/graphene interface [25]. Subsequently, holes with a diameter of 2.5 nm each have been made in the graphene layer (a scaling of the real dimensions was mandatory for computational reasons), and the perforated graphene–SiO_2_ interface has been optimized again. At the end of the optimization, the surface was slightly wavy (Figure 10a), with concave areas located in correspondence with the holes (Figure 10b). Globally, the interface system appears like a perforated network (Figure 10c), in which, observing from above, it is possible to see the Si atoms no longer involved in chemical bonds due to the holes themselves (Figure 10d).

**Figure 11:**
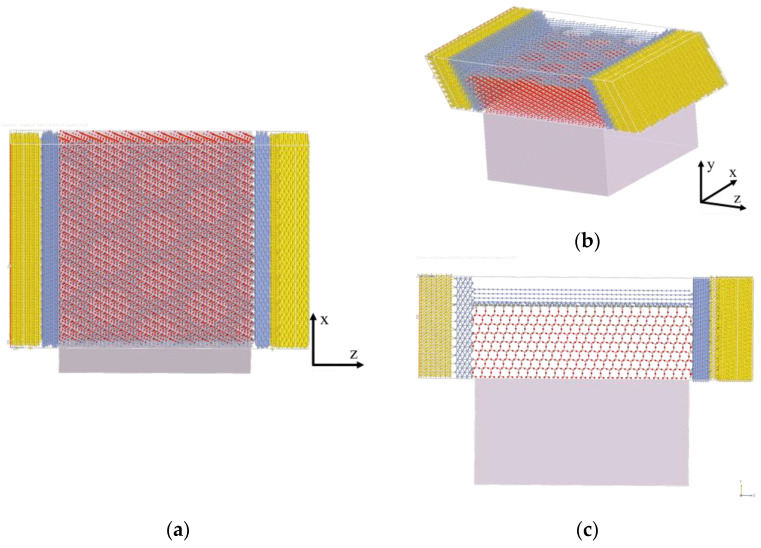
An atomistic description of the “triangle” nanomesh diode: (**a**) top, (**b**) lateral, and (**c**) front views. The C, Si, O, Cr, and Au atoms are highlighted in gray, light brown, red, blue, and yellow, respectively.

Once this interface was identified, the entire device was modeled by placing crystalline Cr on top of the perforated graphene and on the sides of the central area; then, two sections made by Au atoms have been included as metal contacts on the sides of the device, simulating the source and drain. The middle region of the device (excluding Cr and Au) is 10.7 nm long, and below it, an implicit region with a depth of 6 nm has been placed. This implicit region has a value of its real permittivity ε_r_ = 13.8 to mimic the effect of silicon as a bulk substrate. To conclude, the entire device has the following scaled dimensions: x = 11.24 nm, y = 11.72 nm, and z = 14.43 (Figure 11).

**Figure 12:**
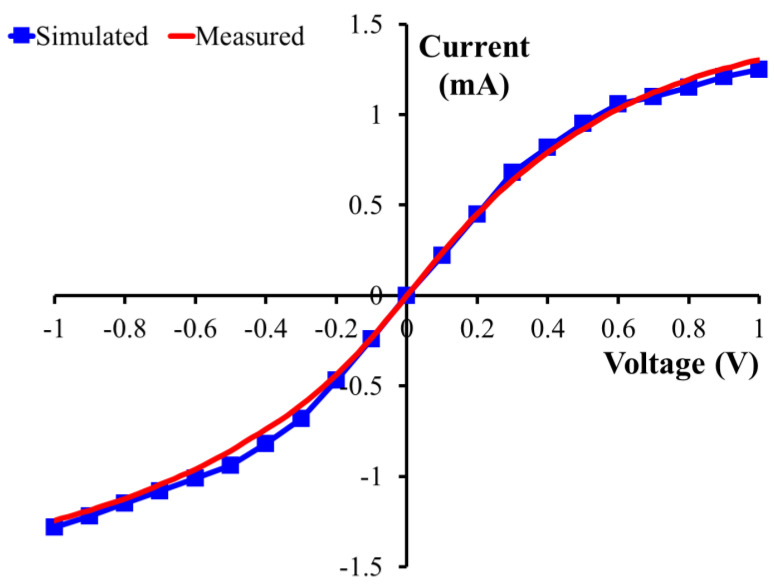
A comparison between the simulated and measured I–V curve of the “triangle” nanomesh diode in the voltage range between −1 and 1 V.

Finally, the I–V characteristic has been calculated by considering 21 points in the whole voltage range between −1 and 1 V (Figure 12). The results of the simulations are superimposed on the measured curve, showing impressive agreement, thus demonstrating the importance of the ab initio approach to predict and/or better understand the electrical behavior of quantum devices based on two-dimensional materials. The excellent agreement also proves that the atomistic description of the interfaces is very accurate, thus allowing us to investigate aspects that might be difficult to observe from an experimental point of view.

## 6. Conclusions

In this paper, we have fabricated at the wafer level and measured tens of graphene nanomesh structures, investigating if these structures are feasible for electromagnetic energy harvesting. The main finding is that a large number of these nano-patterned diodes exhibit excellent properties for harvesting electromagnetic radiation. We stress here that these nano-perforated structures are a part of the so-called graphene nanoporous materials, with excellent absorbing properties at microwave and infrared frequencies. This makes the proposed graphene nanomesh structures ideal candidates as nonlinear devices to be integrated into harvesters at different wavelengths, a subject that will be further investigated in a forthcoming paper. Their fabrication is a key factor in the continuation of this work, the most important issue being the edge roughness effects. These effects are well explained based on transmission electron microscopy (TEM) measurements and DC measurements at 4 K [26]. However, even if the etching is performed with RIE in place of plasma oxygen (as in our case), the effects of EBL itself (i.e., misalignments and proximity) cannot be avoided due to the very small dimensions. Since we are dealing with graphene periodic structures, the devices studied by us are graphene metasurfaces with excellent applications in optics and photonics [27,28,29].

## Data Availability

The original contributions presented in this study are included in the article; further inquiries can be directed to the corresponding author.
